# Prognostic and Predictive Factors in Advanced Urothelial Carcinoma Treated with Immune Checkpoint Inhibitors: A Review of the Current Evidence

**DOI:** 10.3390/cancers13215517

**Published:** 2021-11-03

**Authors:** Sara Elena Rebuzzi, Giuseppe Luigi Banna, Veronica Murianni, Alessandra Damassi, Emilio Francesco Giunta, Filippo Fraggetta, Ugo De Giorgi, Richard Cathomas, Pasquale Rescigno, Matteo Brunelli, Giuseppe Fornarini

**Affiliations:** 1 Medical Oncology, Ospedale San Paolo, 17100 Savona, Italy; 2 Department of Internal Medicine and Medical Specialties (Di.M.I.), University of Genova, 16132 Genova, Italy; 3 Candiolo Cancer Institute, FPO-IRCCS, 10060 Candiolo, Italy; gbanna@yahoo.com; 4 Medical Oncology Unit 1, IRCCS Ospedale Policlinico San Martino, 16132 Genova, Italy; murianni.veronica@gmail.com (V.M.); giuseppe.fornarini@hsanmartino.it (G.F.); 5 Academic Unit of Medical Oncology, IRCCS Ospedale Policlinico San Martino, 16132 Genova, Italy; alessandra.damassi@gmail.com; 6 Department of Precision Medicine, Università Degli Studi della Campania Luigi Vanvitelli, 80131 Naples, Italy; emiliofrancescogiunta@gmail.com; 7 Pathology Department, Ospedale “Gravina”, 95041 Caltagirone, Italy; filippofra@hotmail.com; 8 Department of Medical Oncology, IRCCS Istituto Romagnolo per lo Studio dei Tumori (IRST) “Dino Amadori”, 47014 Meldola, Italy; ugo.degiorgi@irst.emr.it; 9 Division of Oncology/Hematology, Kantonsspital Graubünden, 7000 Chur, Switzerland; richard.cathomas@ksgr.ch; 10 Interdisciplinary Group for Translational Research and Clinical Trials, Urogenital Cancers GIRT-Uro, Candiolo Cancer Institute, FPO-IRCCS, Candiolo, 10060 Turin, Italy; pasquale.rescigno@ircc.it; 11 Department of Diagnostics and Public Health, Section of Pathology, University and Hospital Trust of Verona, 37134 Verona, Italy; matteo.brunelli@univr.it

**Keywords:** advanced urothelial carcinoma, immune checkpoint inhibitor, immunotherapy, prognostic, biomarkers, tumour mutational board, genomic signatures, ctDNA, PD-L1, inflammatory indices

## Abstract

**Simple Summary:**

Despite immune checkpoint inhibitors’ (ICIs) improved overall survival in urothelial carcinoma patients, only a minority of them benefit from immunotherapy. Therefore, there is an unmet clinical need to identify biomarkers which are useful to select the patients who are most likely to respond to ICIs. This review describes the prognostic and predictive role, and potential clinical applicability, of patient- and tumour-related factors. These factors include new molecular classes, tumour mutational burden, mutational signatures, circulating tumour DNA, programmed death-ligand 1, inflammatory indices and clinical characteristics. This summary may help clinicians to assess patients who are considered for ICI treatment, and may drive further prospective research on these biomarkers.

**Abstract:**

In recent years, the treatment landscape of urothelial carcinoma has significantly changed due to the introduction of immune checkpoint inhibitors (ICIs), which are the standard of care for second-line treatment and first-line platinum-ineligible patients with advanced disease. Despite the overall survival improvement, only a minority of patients benefit from this immunotherapy. Therefore, there is an unmet need to identify prognostic and predictive biomarkers or models to select patients who will benefit from ICIs, especially in view of novel therapeutic agents. This review describes the prognostic and predictive role, and clinical readiness, of clinical and tumour factors, including new molecular classes, tumour mutational burden, mutational signatures, circulating tumour DNA, programmed death-ligand 1, inflammatory indices and clinical characteristics for patients with urothelial cancer treated with ICIs. A classification of these factors according to the levels of evidence and grades of recommendation currently indicates both a prognostic and predictive value for ctDNA and a prognostic relevance only for concomitant medications and patients’ characteristics.

## 1. Introduction

Worldwide, urothelial carcinoma (UC) represents the seventh most common cancer and the ninth most deadly tumour, with about 212,000 related deaths [1].

For a long time, the only effective treatment of metastatic UC (mUC) was platinum-based chemotherapy, which is still the standard of care in the first-line setting [2]. In recent years, the treatment landscape of mUC has been changed profoundly by the introduction of immune-checkpoint inhibitors (ICIs) [3] and, even more recently, antibody–drug conjugates (anti-nectin 4 and anti-Trop2) [4] and FGFR inhibitors [5]. 

Since 2016, the U.S. Food and Drug Administration (FDA) has approved two monoclonal antibodies targeting PD-1 (nivolumab and pembrolizumab) and three antibodies targeting PD-L1 (atezolizumab, avelumab, and durvalumab) for mUC [6]. 

The introduction of these new drugs urges the identification of potential biomarkers which are able to select the patients most likely to respond to immunotherapy. Promising prognostic and predictive factors in patients with mUC treated with ICIs include clinical features, new tumour molecular classes, the tumour mutational burden (TMB), mutational signatures, circulating tumour DNA (ctDNA) and programmed death-ligand 1 (PD-L1). Despite the increasing number of biomarkers under investigation, these factors still need validation for their application in clinical practice.

In this review, we summarize the landscape of clinical, molecular and genomic determinants of the prognosis and response to ICIs in patients with mUC. A classification of these factors by their prognostic and predictive value according to levels of evidence and grades of recommendation [7] based on the available evidence has been attempted.

## 2. Molecular Factors

### 2.1. Molecular Classes

Muscle-invasive bladder cancer (MIBC) is a heterogeneous disease characterized by genomic instability and a high mutation rate [8]. In this scenario, transcriptome profiling may be helpful with the classification of UC into molecular subtypes in order to stratify the prognosis more precisely and drive more effective therapeutic choices. Indeed, the assumption for a molecular tumour classification is the understanding of cancer biology by identifying the specific genomic alterations of which the molecular subtypes are enriched, and which could be clinically significant as prognostic and druggable [9].

Several molecular classifications have been attempted for UC, advancing our knowledge about its biology [9]. The response to chemotherapy and immunotherapy may be enriched in specific MIBC subtypes [10,11,12,13]. However, the diversity of their subtype sets, so far, has impeded their clinical application.

Based on the transcriptomic profiles of 1750 MIBCs from 16 published datasets, two additional cohorts, and a network-based analysis of six independent MIBC classification systems, a consensus set of six molecular classes has been identified by a single-sample transcriptomic classifier [9]. The six molecular classes included: the basal/squamous class (Ba/Sq), accounting for 35% of all MIBC; the luminal/papillary class (LumP), accounting for 24%; the luminal unstable class (LumU), accounting for 15%; the stroma-rich class, accounting for 15%; the luminal non-specified class (LumNS), accounting for 8%; and the neuroendocrine (NE)-like class, accounting for 3% [9]. 

An association of some of these classes with The Cancer Genome Atlas (TCGA) PanCancer clusters has been observed between the Ba/Sq and the Squamous cell carcinoma (C27: Pan-SCC pan-cancer cluster) (*p* < 0.001), and the stroma-rich class with the stroma-driven class (C20: Mixed stromal/immune) (*p* < 0.001) [9]. 

The six molecular classes differ by their underlying oncogenic mechanisms, infiltration by immune and stromal cells, histological and clinical characteristics, and survival outcomes. 

Regarding their prognostic value, based on 872 patients, and taking the LumP class as the reference for class-based survival, the LumNS (hazard ratio [HR] 1.07, 95% confidence interval [CI] 0.63–1.82) and stroma-rich classes (HR 0.98, 95% CI 0.65–1.49) showed a similar prognosis to the LumP class; the LumU class did not have significantly inferior overall survival (OS) (HR 1.49, 95% CI 0.93–2.39), the Ba/Sq class had a significantly poorer prognosis (HR 1.83, 95% CI 1.30–2.58, *p* < 0.001), and the NE-like class had the worst prognosis (HR 2.34, 95% CI 1.09–5.05, *p* < 0.03) [9].

In addition to common urothelial differentiation signatures, like the PPARG/GATA3/FOXA1-related Lund urothelial ones, the luminal classes may selectively bear gene alterations which are potentially relevant as drug targets, such as the FGFR3 in the LumP, and the TP53, high TMB and ERCC2—which are related to higher cell cycle activity and genomic instability—in the LumU. Similarly, the Ba/Sq was enriched in cytotoxic lymphocytes (CTL) and NK, alongside EGFR mutations, the Stroma-rich in T- and B-Cells, and the NE-like in TP53 and RB1 alterations [9].

Concerning treatment responses, an enrichment in responders to atezolizumab was observed among LumNS, LumU and NE-like [9]. However, previous discordant results from the TCGA and different ICIs [11,14] indicate the need for prospective validation. 

The molecular classification might be used for prognostication to assist treatment evaluation, and, at the same time, for a more productive collection of clinical information. However, prospective validation is warranted because the use of such classification is only supported by retrospective clinical data lacking the complete patients’ treatment history. Furthermore, it might represent a robust framework enabling the testing and validation of predictive biomarkers in future prospective clinical trials, including basket trials, based on similarities with other cancer molecular subtypes according to the PanCancer Atlas, such as the Ba/Sq and Lum. 

On the other hand, an integrative multi-omics analysis has recently been performed to better characterize Non-Invasive Muscle Bladder Cancer (NIMBC) [15]. In this study, the authors identified four molecular classes reflecting the behaviour and aggressiveness of UC, driving the implementation of biomarkers with predictive and prognostic value [15].

The genomic landscape of NMIBC showed complex genomic patterns, with activating mutations in FGFR3 and PIK3CA, such as chromosome 9 deletions in early disease [16,17]. The NMIBCs were also subdivided into different progression risk groups based on mutations in FGFR3, the methylation of GATA2 and copy number alterations (CNAs). All of these data could provide new molecular therapeutic targets [16,17].

### 2.2. Tumour Mutational Burden (TMB)

TMB can be defined as the total number of non-synonymous mutations per coding area of a tumour genome [18]. These mutations can be transcribed and translated to generate neoantigens displayed on the cell surface; T-lymphocytes can recognise some of these neoantigens and promote the apoptosis of tumour cells [18]. Tumours with a high mutational load are more likely to express neoantigens, and to induce a strong immune reaction [19]. Several studies have demonstrated an association between a high TMB and the response to immunotherapy in different locally advanced or metastatic solid tumour types [20]. 

In June 2020, the FDA approved pembrolizumab for the treatment of adult and paediatric patients with unresectable or metastatic solid cancers and a high TMB [>10 mutations/megabase (mut/Mb)] [21]. This approval was based on the efficacy data from the phase II KEYNOTE-158 trial, which demonstrated an association between a high TMB and the tumour response to immunotherapy, with a durable response (>2 years) rarely being observed in heavily pre-treated metastatic cancers [22].

UC, along with melanoma and lung cancer, is characterized by a high number of somatic mutations and, therefore, a significant genomic instability [8]. The exploratory analyses of the phase II IMvigor210 trial included the quantification of the mutational load and a correlation with the clinical outcomes [23]. As expected, the TMB was significantly higher in patients who responded to atezolizumab than in non-responders. Moreover, patients with a higher mutational load had significantly longer overall survival than patients with a lower load [23]. 

However, the TMB status alone was not able to stratify the patients according to the survival benefit. In this regard, exploratory analyses of the JAVELIN Bladder 100 trial demonstrated that some DNA mutational signatures, involving certain base pair alterations, were associated with a survival benefit from avelumab, while other mutations were not [24]. This suggests that the type and the location of mutations may influence the predictive role of TMB assessment more accurately than the degree of the mutational load. 

The predictive value of TMB for the pathological response to immunotherapy has also been explored in the neoadjuvant setting [25,26]. In patients with MIBC, a high TMB was generally observed in responders versus non-responders to neoadjuvant pembrolizumab, irrespective of the histological subtypes [27].

The predictive role of combining TMB with PD-L1 was also investigated. Patients with high TMB and PD-L1-positive tumours were more likely to derive a survival benefit from avelumab maintenance therapy [24]. Similar findings were observed in the randomised phase III IMvigor130 study [28]. The association of high PD-L-1 (>5% of immune cells) and a high TMB (>10 mut/MB) was associated with improved OS in the atezolizumab monotherapy arm, as compared to the chemotherapy arm [28]. Nevertheless, similar outcomes were not seen with the combination of atezolizumab and chemotherapy compared to the chemotherapy alone, suggesting a potentially distinct biology driving the benefit from atezolizumab and its combination with chemotherapy, and eventually highlighting the predictive inconsistency of this biomarker combination [28].

As for other tumour types, many factors hinder the clinical application of TMB as a biomarker, including the variability and the lack of a validated cut-off, a clear prognostic value, the differences related to the sequencing platforms used for its assessment, and the high scoring failure rate due to the quantity and quality of tumour tissue analysed [29]. As an example for the mUC, in the IMvigor210 study, the cut-off of the TMB varied between the two different cohorts of cisplatin-ineligible mUC treated with first-line atezolizumab and the platinum-treated patients [23]. Moreover, a high tumour mutational load may not be a specific biomarker for immunotherapy, as it has also been associated with the tumour response to neoadjuvant chemotherapy [30,31].

### 2.3. Mulecular Signatures

The increasing interest in tumour molecular features and new omics technologies has led to the discovery of different molecular signatures. These involve genes, messenger ribonucleic acids (mRNAs) and proteins which are studied as biomarkers to better predict clinical outcomes, or to better understand the cancerogenesis process [32,33].

Furthermore, because the TMB and/or the PD-L1 expression did not precisely identify patients who were more likely to derive benefit from immunotherapy [34,35], the assessment of tumour-related or immune-related gene signatures was actively investigated.

The DNA and RNA sequencing analyses of the IMvigor130 trial investigated the apolipoprotein B editing catalytic polypeptide (APOBEC) signature [28]. The APOBEC enzymes are a family of cytidine deaminases involved in the DNA repair processes, and are responsible for a mutation signature (TCW > T/G) frequently observed in MIBCs [36,37]. Patients with a high APOBEC mutational signature had a longer survival, whether in the atezolizumab monotherapy or the combination arm with chemotherapy compared to chemotherapy alone, whereas a high TGFβ signature predicted a worse OS with the immunotherapy alone [28].

RNA-based immune-gene expression profiling has the advantage of providing information from many cancer cells and immune cells, identifying more accurately the inflammatory status. The Checkmate-275 study investigated an interferon-gamma (IFN-γ) expression signature, and found a significant correlation with the response to nivolumab in the metastatic setting [38].

At the ESMO 2020 meeting, an exploratory analysis of the Javelin Bladder 100 study on tumour biomarkers was presented. While neither PD-L1 and TMB alone nor in combination predicted the response to immunotherapy and survival benefit, the expression of the immune-related genes of both the innate and adaptive immune system (i.e., CD8, IFNG, LAG3, TIGIT and CXCL9) and the number of alleles encoding high-affinity Fc gamma receptor variants predicted the survival benefit from avelumab first-line maintenance [24]. The application of the T-cell-inflamed and JAVELIN-Immuno signatures seemed to correlate with the treatment response with HRs of 0.55 and 0.49, respectively. Furthermore, the JAVELIN-Immuno high signature showed enrichment in some signalling pathways (Notch, Hedgehog, TGFbeta) which were likely to have an increased antitumour response to immunotherapy [24]. 

In the PURE-01 study, investigating the efficacy of pembrolizumab as a neoadjuvant therapy, a T-cell-inflamed signature was able to identify those patients who achieved pathological T0 downstaging. In a subsequent analysis, other RNA-based immune signatures were evaluated for their association with pCR and a high Immune190 signature, as well as hallmark signatures for interferon gamma and interferon-alpha, which were significantly associated with pCR and progression-free survival (PFS) after pembrolizumab in the PURE01 study [27]. Similarly, a correlation between the tumour response to atezolizumab and the transcriptional signature of eight genes (IFNG, CXCL9, CD8A, GZMA, GZMB, CXCL10, PRF1 and TBX21) representing interferon signalling, and the presence of CD8+ effector T cells, namely the tGE8, was reported within the ABACUS neoadjuvant study [39]. 

Moreover, a strong immune-mediated adaptive resistance was observed in non-responding tumours, suggesting the investigation of ICI combinations to counteract the expression of negative regulators of the immune response [40].

The BISCAY study combined durvalumab (anti-PD-L1) with different targeted therapies depending on tumour gene alterations determined by NGS [41]. Despite the overall negative results, this study suggested a negative predictive role for the FGFR expression with regards to immunotherapy. In fact, FGFR-mutated tumours did not have a high expression of immune-active T-cell signatures, and the addition of durvalumab did not result in enhanced activity from the targeting agent only [9,41].

Although the above data look promising, there is a need to standardize molecular assays and find a molecular panel which is applicable to daily clinical practice.

### 2.4. ctDNA

The sequencing of the tumour fraction of the cell-free DNA (ctDNA) is an emerging and sensitive method to detect residual disease, anticipate relapse, and monitor the therapeutic efficacy in patients with several cancer types [42,43]. Furthermore, it provides a basis for clinical studies evaluating early therapeutic interventions [42,43]. 

In UCs, proof-of-concept data documented that ctDNA is detectable in plasma and urine, and could be a prognostic factor [44,45].

Vandekerkhove et al. demonstrated that ctDNA profiling may also identify putative biomarkers for therapy response in bladder cancer (such as FGFR3, ERCC2, ERBB2 and TMB), and represents a cost-effective and minimally invasive method for their identification and patient stratification [46]. Furthermore, the serial monitoring of ctDNA can optimize the use of therapies, as ctDNA fractions are expected to decrease in patients responding to treatment [46].

In a longitudinal analysis, the presence of ctDNA—assessed by whole-exome sequencing (WES) in 68 patients with localised advanced bladder cancer at diagnosis—during chemotherapy, before and after cystectomy and during surveillance, resulted in being highly prognostic at diagnosis (HR 29.1) [47]. The presence of ctDNA identified all of the patients with metastatic relapse (with 100% sensitivity and 98% specificity), with a median of 96 days of diagnostic anticipation compared to the radiographic imaging [47]. Furthermore, the dynamics of ctDNA during chemotherapy, for those patients with positive ctDNA before or during the treatment, were associated with disease recurrence. It is worth noting that pathological downstaging, including related mutational signatures, was not associated with disease recurrence [47].

A post-surgical ctDNA detection was also associated with a higher risk for recurrence and death in the randomised phase III IMvigor010 study with adjuvant atezolizumab versus observation following cystectomy for patients with MIBC [48]. Moreover, the presence of ctDNA was predictive, as it was able to identify the patients who were likely to benefit from adjuvant atezolizumab. The IMvigor010 study did not meet his primary endpoint of disease-free survival (DFS) in the overall population of 809 enrolled patients [48]. The ctDNA was assessed by WES at a median of 11 weeks post-cystectomy in 581 patients with evaluable samples, comprising 72% of the intent-to-treat population of the IMvigor010 study. While the detection of ctDNA (in 214 patients, 37%) was associated with worse DFS and OS compared to the absence of ctDNA (in 367 patients, 63%), a significant difference in DFS (HR 0.58, 95% CI 0.43–0.79) and OS (0.59, 95% CI 0.41–0.86, *p* = 0.0059) was observed in favour of the patients with detectable ctDNA treated with atezolizumab compared to the observational arm. Furthermore, in patients with the presence of ctDNA, the rate of ctDNA clearance from cycle 1 to cycle 3 was significantly higher in the atezolizumab arm versus the observational one, with ctDNA clearance rates of 18.2% and 3.8%, respectively (*p* = 0.0048) [48]. 

### 2.5. Programmed Death Ligand-1 (PD-L1)

In the first-line treatment of mUC, a high PD-L1 combined positive score (CPS) of ≥10%, defined as the percentage of tumour cells (TC) and immune cells (IC)/the total tumour cells given by the 22C3 Dako assay, was associated with a prolonged median OS (mOS) in patients ineligible for cisplatin treatment with first-line pembrolizumab in the single-arm phase II KEYNOTE 052 study. The mOS was 18.3 versus 9 months in patients with PD-L1 CPS ≥ 10% and <10%, respectively, and it was 11.3 months in the overall population [49]. As the KEYNOTE 052 was not a controlled phase III study, a favourable prognostic value only for the high PD-L1 expression could not be rule out. Indeed, in the following KEYNOTE 361, IMvigor130 and Danube phase III randomised trials, the anti-PD1/PD-L1 agents either alone or in combination with chemotherapy were not superior to the chemotherapy according to OS in patients with high PD-L1 tumours, as well as in the overall population [50,51,52]. Only in the Danube trial did the combination of the anti-PD-L1 inhibitor durvalumab and anti-CTLA4 tremelimumab show a superior OS compared to chemotherapy in patients with high PD-L1. Therefore, the predictive value of high PD-L1 remains uncertain [52]. In contrast, a decreased survival with first-line pembrolizumab or atezolizumab compared to platinum-based chemotherapy was reported by the KEYNOTE 361 and IMvigor130 phase III trials for patients with low PD-L1 tumour expression, suggesting a negative predictive value for low PD-L1 expression [50,51]. A low PD-L1 expression was defined in the KEYNOTE 361 and IMvigor130 studies as a CPS < 10% or positive IC < 5% by the immunohistochemistry (IHC) 22C3 pharmDx and SP142 Ventana assays, respectively [50,51].

In the maintenance setting of mUC, following first-line chemotherapy, the Javelin-100 phase III trial showed an advantage in OS from the anti-PD-L1 avelumab compared to the best supportive care irrespective of the positive PD-L1 expression, which was assessed by a SP263 Ventana assay and classified as positive if at least one of the following three criteria were met: at least 25% TC staining for PD-L1, at least 25% IC staining for PD-L1 if more than 1% of the tumour area contained IC, or 100% ICs staining for PD-L1 if no more than 1% of the tumour area contained ICs [53].

In the adjuvant setting of UC, the IMvigor 010 with the anti-PD-L1 atezolizumab did not show a significant advantage in DFS compared to observation either in patients with positive PD-L1 tumours IC according to the SP142 Ventana assay, or in the overall population [54], while the Checkmate274 showed a significant DFS advantage in favour of nivolumab versus observation in the intention-to-treat population with a better HR in patients with a PD-L1 expression ≥ 1% (HR 0.55 vs. 0.70 for the overall population), as assessed on TC by a PD-L1 IHC 28-8 pharmDx assay [55]. 

In the second-line of mUC, in the IMvigor 211 phase III study with anti-PD-L1 atezolizumab compared to chemotherapy, the OS was not significantly different in patients with a high PD-L1 defined by IC 2/3 (or ≥5%/10% of PD-L1 positive IC according to a SP142 Ventana assay) [56]. In contrast, in the KEYNOTE 045 study, a significant difference in OS was observed in favour of the anti-PD-1 pembrolizumab versus chemotherapy in patients with high PD-L1 tumours defined as CPS according the 22C3 Dako assay [57]. Although possible differences in efficacy between the anti-PD1 and anti-PD-L1 agents cannot completely be ruled out [58], plausible explanations may rely on the different tests and scores used to assess the PD-L1 expression, as well as on the tumoural site and timing of the biopsy in respect to the disease history. In this regard, it is noteworthy that the mOS duration in the high PD-L1 subgroup of patients differed between those two trials despite the similar populations enrolled and the mOS observed in the overall population of the chemotherapy control arm (of 8.4 and 7.3 months, for the IMvigor211 and the Keynote-045 study, respectively) [56,57]. In the KEYNOTE 045 study, the mOS of patients with high CPS (≥10%) treated with pembrolizumab was of 8.0 months, which was significantly longer than 5.2 months with chemotherapy (*p* = 0.0048) [57]; on the other hand, in the IMvigor211 study, the patients with high IC 2/3 had an mOS of 11.1 months with atezolizumab, which was not significantly longer than 10.6 months with chemotherapy (*p* = 0.41) [56]. The OS difference seen in the chemotherapy control arms of these two studies, with a different definition of high PD-L1 tumours, might suggest a positive prognostic value which is marginally predictive for the high PD-L1 by the IC 2/3, and a negative prognostic—although predictive—value by the CPS. 

In the neoadjuvant setting, only phase II studies are currently available. However, the Pure-01 study demonstrated the dynamic characteristic of the PD-L1 by showing its significantly increased expression as CPS following three administrations of the anti-PD1 pembrolizumab [40]. 

In conclusion, the current evidence on the prognostic and predictive value of PD-L1 expression is limited by the different diagnostic assays used in the clinical trials for each anti-PD1 or anti-PD-L1 agent, and remains inconclusive. PD-L1 may have a prognostic role, the value of which may also change depending on the type of tumour cells scored (i.e., negative if CPS, or positive if IC), and may be predictive only for the low expression. 

The molecular predictive and prognostic factors in UCs are summarized in Table 1. 

## 3. Clinical Factors

### 3.1. Patient’s Characteristics 

Several clinical factors have been reported to be prognostic in patients with mUC treated with chemotherapy. The most important ones include performance status (PS), metastatic sites, haemoglobin levels, and time from prior chemotherapy. Bajorin et al. showed that, in untreated patients, a poor Karnofsky Performance Status (KPS) < 80% and the presence of visceral (liver, lung, and bone) metastases were independent prognostic factors associated with a worse OS [59]. Bellmunt et al. reported that a poor Eastern Cooperative Oncology Group (ECOG) PS ≥ 2, low haemoglobin levels (<10 g/dL) and the presence of liver metastasis were independent poor prognostic factors in patients who failed platinum-based chemotherapy [60]. Moreover, a shorter time from prior chemotherapy (<3 months) has been reported to enhance the prognostic classification of patients receiving second-line therapy [61].

The survival benefit of immunotherapy in both untreated and pretreated patients with mUC has been observed regardless of all of those clinical factors, confirming their prognostic but not predictive value, in many retrospective analyses [62,63,64,65,66,67,68,69,70] and subgroup and post-hoc analyses of prospective randomised trials [71,72].

### 3.2. Concomitant Medications

Several retrospective analyses and meta-analyses have recently been reported in the literature about the impact of concomitant medications on the clinical outcomes of patients with cancer treated with ICIs [73,74,75,76,77,78]. These studies mainly included patients with non-small cell lung cancer (NSCLC), melanoma or renal cell carcinoma (RCC). Commonly used drugs in clinical practice—such as corticosteroids, antibiotics and proton pump inhibitors (PPIs)—have been reported to negatively affect the activity of immunotherapy through immune-modulatory effects. In fact, these drugs may induce a detrimental effect on the immune system and gut microbiota, which is a well-known regulator of immune homeostasis [79]. A drug-based prognostic score developed by Buti et al.—including the cumulative exposure to high-dose steroid therapy (a dose of ≥ 10 mg prednisone-equivalents per day), antibiotics and PPIs—can help to stratify patients treated with ICIs in routine practice and clinical trials [73].

The post-hoc analyses of the single-arm phase II IMvigor210 trial and the randomised phase III IMvigor211 trial confirmed the negative predictive role of PPI and antibiotic use in patients with mUC treated with ICI, but not in the case of treatment with chemotherapy [80,81]. 

### 3.3. Inflammatory Indices

An inflammatory condition in patients with cancer has been associated with worse outcomes and a lower therapeutic response across different tumour types [82]. Inflammatory indices from peripheral blood have been investigated as potential biomarkers in different tumours, settings and therapies [83,84,85], including patients with UC treated with surgery or chemotherapy [64,86,87,88]. 

As far as ICIs are concerned, inflammatory indices have been mostly studied in patients with advanced NSCLC and melanoma [89,90], while fewer and smaller studies have been conducted in patients treated with ICIs for genitourinary tumours, including UC [62,63,65,69,70,91,92,93,94,95,96,97]. The inflammatory indices most commonly studied in patients with UC treated with ICIs include the neutrophil-to-lymphocyte ratio (NLR), baseline platelet-to-lymphocyte ratio (PLR), lymphocyte-to-monocyte ratio (LMR), lactate dehydrogenase (LDH), C-reactive protein (CRP) and albumin. High levels of NLR, PLR, LDH and CRP, and low levels of albumin have been correlated with worse survival and efficacy outcomes, either as single parameters or within combined prognostic scores [62,63,65,69,70,92,93,94,95,96,97,98]. 

### 3.4. Combined Prognostic Tools 

Inflammatory indices from peripheral blood have been investigated in combination with other clinical prognostic factors within prognostic models for risk-stratification in several cancer types treated with ICIs, especially the NSCLC [91,98,99,100]. The interest in prognostic models has also recently been increasing for genitourinary tumours, including RCC [101,102,103] and UC [63,65,68,95,96,97,104].

In patients with mUC treated with ICIs, the most frequently included factors in prognostic models are inflammatory indices (such as NLR, C-reactive protein and albumin) and pretreatment clinical parameters (i.e., the PS and metastatic site) [63,65,68,95,96,97,104,105,106]. The molecular factors included in the prognostic scores were PD-L1 expression and genomic parameters (e.g., single-nucleotide variants) [96,104]. 

Most of these analyses are derived from retrospective studies, except for the two prognostic scores developed by Fornarini et al. and Sonpavde et al., and the machine learning (ML) model developed by Abuhelwa et al. [104,105,106]. The two prognostic models were developed by the retrospective analysis of the phase IIIb SAUL trial [104] and the two phase I/II trials [105], both evaluating second-line ICI monotherapy. The ML model was built using the atezolizumab cohort of the IMvigor210 trial as the training cohort, and the IMvigor211 trial population as the external validation [106].

Most of these prognostic models (Table 2) need external validation with prospective studies before being incorporated into clinical practice; only one of them has shown a predictive value for immunotherapy but not for chemotherapy in a retrospective analysis [96].

## 4. Radiomics

Due to the growing need to identify useful biomarkers to select the patients who are most likely to benefit from ICIs, the quantitative analysis of imaging features by artificial intelligence algorithms, namely radiomics, has recently been investigated as a possible surrogate marker to predict the outcome of patients treated with immunotherapy [107,108]. Radiomics has been reported as a promising approach to predict the response and survival outcomes in patients with NSCLC and melanoma receiving ICIs [109,110]. Three retrospective analyses investigated radiomics from baseline contrast-enhanced computed tomography (CT) images in patients with mUC receiving anti-PDL1 or anti-PD1 monotherapy [111,112,113]. 

Artificial intelligence algorithms may also allow us to combine the information obtained by the image features with other clinical and laboratory prognostic factors, thus increasing the diagnostic accuracy of predictive models [112,113]. 

Artificial intelligence and deep machine learning-based models may provide helpful decision tools to clinicians for the selection of patients for ICIs in the near future. However, none of these radiomics-based models have been assessed in patients with UC treated with chemotherapy yet; further studies on larger populations and external validation are still needed to confirm their predictive value.

## 5. Conclusions

In the current era, in which ICIs have been tested for several tumour types, it is crucial to identify prognostic and predictive biomarkers or models which are able to select UC patients who will benefit from ICIs, especially as novel therapeutic agents could soon become available in clinical practice for the treatment of UC. In addition, adjuvant ICIs have recently shown a benefit in terms of disease-free survival in patients with radically resected muscle-invasive urothelial carcinoma [112]. With more follow-up, a survival benefit is awaited, opening new methods for biomarker identification even in this setting.

By reviewing the available evidence and attempting a classification of clinical and tumour factors according to levels of evidence and grades of recommendation (see Table 3), both a prognostic and a predictive value is currently suggested for ctDNA, while only a prognostic relevance seems to apply to concomitant medications and patient’s characteristics. 

We think this information may be helpful to clinically assess mUCs patients who are considered for treatment with ICIs, and could also drive further prospective research on these biomarkers, either as single factors or within combined prognostic models implemented by artificial intelligence algorithms. 

## Figures and Tables

**Table 1:** Molecular factors and their prognostic and predictive value in UC patients, with a particular focus on ICIs.

Category	Description	Prognostic and Predictive Value in UC	Notes
Molecular classes	6 molecular (transcriptomic) classes based on a consensus of MIBC: Squamous (Ba/Sq)-35%; luminal/papillary (LumP), 24%; luminal unstable (LumU), 15%; stroma-rich, 15%; luminal non-specified (LumNS), 8%; neuroendocrine (NE)-like, 3% [9].	Ba/Sq is associated with shorter OS, NE-like is associated with worse prognosis (LumP as reference) [9]. LUmNS, LumU, NE-like are more responsive to ICIs [9].	Need for prospective validation
TMB	Total number of non-synonymous mutations per coding area of a tumour genome. UC is characterized by high values of TMB compared to other tumours [23].	High TMB predicts OS benefit from avelumab maintenance therapy [23] and improved OS with atezolizumab when compared to CT [28]. TMB was higher in responder to neoadjuvant pembrolizumab [27].	Issues: variability, lack of a validated cut-off, differences related to the sequencing platforms
Molecular signatures	Study of involved genes (DNA sequencing), messenger ribonucleic acids (mRNAs) (RNA sequencing), and proteins (transcriptome) in tumor samples.	APOBEC mutational signature predicts OS benefit with atezolizumab ± CT compared to CT alone [28]. TGF-β signature predicts worse OS with ICIs [28]. IFN-γ signature correlates with response to nivolumab [38]. JAVELIN -Immuno high signature correlates with increased responses to ICIs [24]. Transcriptional signature of eight genes (IFNG, CXCL9, CD8A, GZMA, GZMB, CXCL10, PRF1 and TBX21) correlates with response to atezolizumab [39]. FGFR mutations predict low response to durvalumab [41].	Need to standardize molecular assays and find a molecular panel applicable to daily clinical practice
ctDNA	Quantitative and qualitative analysis of circulating tumoral DNA detected on blood samples.	ctDNA detection is associated with worse prognosis in early stages and could identify metastatic relapses before imaging [47]. ctDNA detection is predictive for adjuvant atezolizumab (both DFS and OS) [48]. ctDNA profiling may be predictive for response to specific therapies [46].	
PD-L1	Expression of the ligand of PD1 receptor has been widely studied as predictive biomarker of response to anti-PD1 and anti-PD-L1 therapies across human cancers.	Possibly predictive for anti-PD1 and anti-PD-L1 agents used in UC patients as adjuvant [54], first-line [49], maintenance after first-line [53] and second-line therapy [56].	Issues: different diagnostic assays used in clinical trials for each anti-PD1 or anti-PD-L1 agent, discordant efficacy of these agents between studies

Abbreviations: UC, urothelial carcinoma; ICIs, immune checkpoint inhibitors; TMB, tumor mutational burden; OS, overall survival; CT, chemotherapy; ctDNA, circulating tumor DNA.

**Table 2:** Clinical factors and their prognostic and predictive value in UC patients, with a particular focus on ICIs.

Category	Description	Prognostic and Predictive Value in UC
Patient’s characteristics	Performance status (PS), metastatic sites, haemoglobin levels are prognostic factors in human cancer.	Karnofsky PS < 80%, ECOG PS ≥ 2, low haemoglobin levels and the presence of visceral metastases are associated with worse OS in mUC patients treated with CT [59,61]. Prognostic but not predictive in mUC patients treated with ICIs [62,63,64,65,66,67,68,69,70,71,72].
Concomitant medications	Commonly used drugs in clinical practice may affect clinical outcomes of cancer patients treated with ICIs.	Use of antibiotics and PPIs have a negative predictive role in mUC patients treated with ICIs but not in those treated with CT [80,81].
Inflammatory indices	Inflammatory indices, like NLR, CMP and albumin, have been investigated in several human cancer types as prognostic tools, especially regarding ICIs.	High levels of NLR, PLR, LDH, CRP and low levels of albumin have been correlated with worse survival and efficacy outcomes in mUC patients [62,63,64,70,92,93,94,95,96,97,98].
Combined prognostic tools	Combinations of inflammatory indices and clinical factors have been investigated in several human cancer types as prognostic tools, especially regarding ICIs.	Two prognostic models were developed in second-line ICIs mono-therapy [104,105]. A machine learning model was built from IMvigor210 atezolizumab arm and validated in IMvigor211 atezolizumab arm [106].

Abbreviations: UC, urothelial carcinoma; mUC, metastatic urothelial carcinoma; ICIs, immune checkpoint inhibitors; OS, overall survival; CT, chemotherapy; PPIs, proton pump inhibitors; NLR, neutrophil-to-lymphocyte ratio; PLR, platelet-to-lymphocyte ratio; LMR, lymphocyte-to-monocyte ratio; LDH, lactate dehydrogenase; CRP, C-reactive protein (CRP).

**Table 3:** Prognostic and predictive values of tumour and clinical factors for their impact on the response and survival outcomes in mUC treated with ICIs.

Variable	Prognostic			Predictive		
Parameter	Clinical Value	Strenght of Evidence	Outcome Variable	Clinical Value	Strenght of Evidence	Outcome Variable
**Tumour molecular factors**						
Molecular classes for MIBC	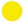	IV, B	PFS, OS	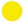	IV, B	PFS, OS
TMB	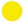	IV, C	ORR, PFS, OS	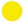	I, C	ORR, PFS, OS
Mutational signatures ^1^	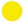	IV, C	ORR, PFS, OS	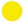	I, C	ORR, PFS, OS
ctDNA	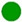	II, A	DFS, OS	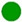	II, A	DFS, OS
PD-L1	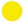	I, C	DFS, PFS, OS	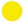	I, B ^2^	DFS, PFS, OS
**Clinical factors**						
Patient’s characteristics ^3^	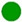	I, A	ORR, PFS, OS	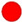	I, A	ORR, PFS, OS
Concomitant medications ^4^	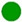	I, A	ORR, PFS, OS	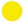	I, B	ORR, PFS, OS
Inflammatory indices ^5^	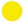	IV, A	ORR, PFS, OS	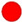	IV, A	ORR, PFS, OS
Combined tools ^6^	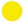	III, B	ORR, PFS, OS	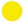	IV, C	ORR, PFS, OS
**Radiomics**						
Radiomics-based models ^7^	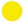	IV, B	ORR, PFS, OS	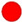	NI	NI

*PFS*, progress-free survival; *OS*, overall survival; *ORR*, overall response rate; *DFS*, disease-free survival; *NI*, not investigated. ^1^ DNA and RNA signatures such as the APOBEC, TGFbeta, IFN-γ and IFN-alpha; immune-related signatures such as the JAVELIN-Immuno or Immune190. ^2^ Evidence was limited due to its low expression. ^3^ Poor PS, visceral metastases, mainly liver metastases, low hemoglobin levels, and a shorter time from prior chemotherapy (<3 months). ^4^ High-dose steroid therapy, antibiotics and PPIs. ^5^ Inflammatory indices from peripheral blood, such as ratios of immune system cells (e.g., NLR) or LDH. ^6^ Combination of inflammatory indices and clinical parameters. ^7^ Radiomic features with/without clinical factors. 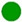 Green circle: clinically useful. 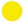 Yellow circle: uncertain clinical usefulness. 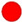 Red circle: not clinically useful. The strength of evidence was adapted from the Infectious Diseases Society of America-United States Public Health Service Grading System [7]. Levels of evidence: I, evidence from at least one large randomised, controlled trial of good methodological quality (low potential for bias) or meta-analyses of well-conducted randomised trials without heterogeneity; II, small randomised trials or large randomised trials with a suspicion of bias (lower methodological quality), or meta-analyses of such trials or of trials with demonstrated heterogeneity; III, prospective cohort studies; IV, retrospective cohort studies or case-control studies; V, studies without a control group, case reports, or expert opinions. Grades of recommendation: A, strong evidence for efficacy with a substantial clinical benefit, strongly recommended; B, strong or moderate evidence for efficacy, but with a limited clinical benefit, generally recommended; C, insufficient evidence for efficacy or benefit does not outweigh the risk or the disadvantages (adverse events, costs, etc.), optional; D, moderate evidence against efficacy or for adverse outcomes, generally not recommended; E, strong evidence against efficacy or for adverse outcomes, never recommended.
